# Global research trends of endoscope in early gastric cancer: A bibliometric and visualized analysis study over past 20 years

**DOI:** 10.3389/fonc.2023.1068747

**Published:** 2023-04-06

**Authors:** Sifan Liu, Nan Zhang, Yan Hao, Peng Li

**Affiliations:** ^1^Department of Gastroenterology, Beijing Friendship Hospital, Capital Medical University, National Clinical Research Center for Digestive Diseases, Beijing Digestive Disease Center, Beijing Key Laboratory for Precancerous Lesion of Digestive Diseases, Beijing, China; ^2^Department of Cardiology, The Fourth Affiliated Hospital of Harbin Medical University, Harbin, China

**Keywords:** early gastric cancer, endoscope, bibliometric analysis, VOSviewer, hotspots

## Abstract

**Objectives:**

Early gastric cancer (EGC) is defined as aggressive gastric cancer involving the gastric mucosa and submucosa. Early detection and treatment of gastric cancer are beneficial to patients. In recent years, many studies have focused on endoscopic diagnosis and therapy for EGC. Exploring new methods to analyze data to enhance knowledge is a worthwhile endeavor, especially when numerous studies exist. This study aims to investigate research trends in endoscopy for EGC over the past 20 years using bibliometric analysis.

**Methods:**

Original articles and reviews examining the use of endoscopy for EGC published from 2000 to 2022 were retrieved from the Web of Science Core Collection, and bibliometric data were extracted. Microsoft Office Excel 2016 was used to show the annual number of published papers for the top 10 countries and specific topics. VOSviewer software was used to generate network maps of the cooccurrences of keywords, authors, and topics to perform visualization network analysis.

**Results:**

In total, 1,009 published papers met the inclusion criteria. Japan was the most productive country and had the highest number of publications (452, 44.8%), followed by South Korea (183, 18.1%), and China (150, 14.9%). The National Cancer Center of Japan was the institution with the highest number of publications (48, 4.8%). Ono was the most active author and had the highest number of cited publications. Through the network maps, exploring endoscopic diagnosis and therapy were major topics. Artificial intelligence (AI), convolutional neural networks (CNNs), and deep learning are hotspots in endoscopic diagnosis. *Helicobacter pylori* eradication, second-look endoscopy, and follow-up management were examined.

**Conclusions:**

This bibliometric analysis investigated research trends regarding the use of endoscopy for treating EGC over the past 20 years. AI and deep learning, second-look endoscopy, and management are hotspots in endoscopic diagnosis and endoscopic therapy in the future.

## Introduction

Gastric cancer is the fifth most commonly diagnosed cancer in the world and the fourth leading cause of cancer-related deaths (1). Although the trend of gastric cancer is currently decreasing, the prevalence is still high in some areas, such as Asia, Eastern Europe, and South America (1, 2). Patients’ quality of life and prognosis are poor. Thus, early detection and treatment of gastric cancer is necessary and fundamental.

Early gastric cancer (EGC) is defined as gastric cancer involved in the gastric mucosa and gastric submucosa layer, with or without lymph node metastasis (3). Endoscopy is widely used in the diagnosis and therapy of EGC, such as endoscopic submucosal dissection (ESD) and endoscopic mucosal resection (EMR) (4). Many studies have focused on the use of endoscopy for EGC in recent years, including etiologies and novel diagnostic and therapeutic methods (5–7). The role of endoscopy in EGC has gradually become more important with the development of numerous technologies, and positive effects have been observed.

Since the start of the 21st century, many researchers and institutions have examined the use of endoscopy for EGC, and many related papers have been published. Hence, it is important to conduct systematic, comprehensive, and scientific analyses to specify the effect of these publications, confirm the hotspots, and identify possible promising fields in this area. Bibliometric and visual analysis can generate network maps based on the co-occurrence of keywords, authors, and topics (8, 9), thereby offering scientific production and development situations in this field (10, 11).

This study aims to perform bibliometric and visualization analysis through VOSviewer software to investigate the global research trends in endoscopy for EGC over the past 20 years and then obtain a better understanding of the current research and situations by analyzing their characteristics.

## Materials and methods

### Data collection

Papers published between 2000 and 2022 were researched in the Web of Science Core Collection (WoSCC) database, which provides more information on scientific publications and was considered the optional database for bibliometric analysis (12). The research strategy was as follows: “((TS = (“early gastric cancer”)) OR TS = (“early gastric carcinoma”)) AND ((TS = (“endoscope”)) OR TS = (“endoscopy”)). The exclusion criteria were as follows: (1) studies not related to endoscopy; (2) studies not written in English; and (3) studies not described in an original article or review. To avoid selection bias, two authors independently searched papers and extracted data on the same day, i.e., 1 August 2022. Disagreements between the authors were resolved through discussion and consensus. The following bibliometric data were extracted: author, title, published year, keywords, and abstract.

### Data analysis

The recorded data were imported into Microsoft Office Excel 2016 and VOSviewer version 1.6.16 software. All papers were analyzed according to their topics: endoscopic diagnosis, endoscopic therapy, etiology, and others (reviews or case reports). Microsoft Office Excel 2016 was used to analyze the annual number of published papers from the top 10 countries and specific topics. VOSviewer version 1.6.16 software (Leiden University, Leiden, Netherlands) was used to generate network maps and cluster visualizations based on the cooccurrence of keywords, authors, and topics (13). In the visualization analysis, each circle represents a keyword or an author, and a larger circle represents a higher frequency of occurrence. Different colors of circles represent different clusters. We used the STARD checklist when writing our report (14).

## Results

### Research trends of publications

**Figure 1:**
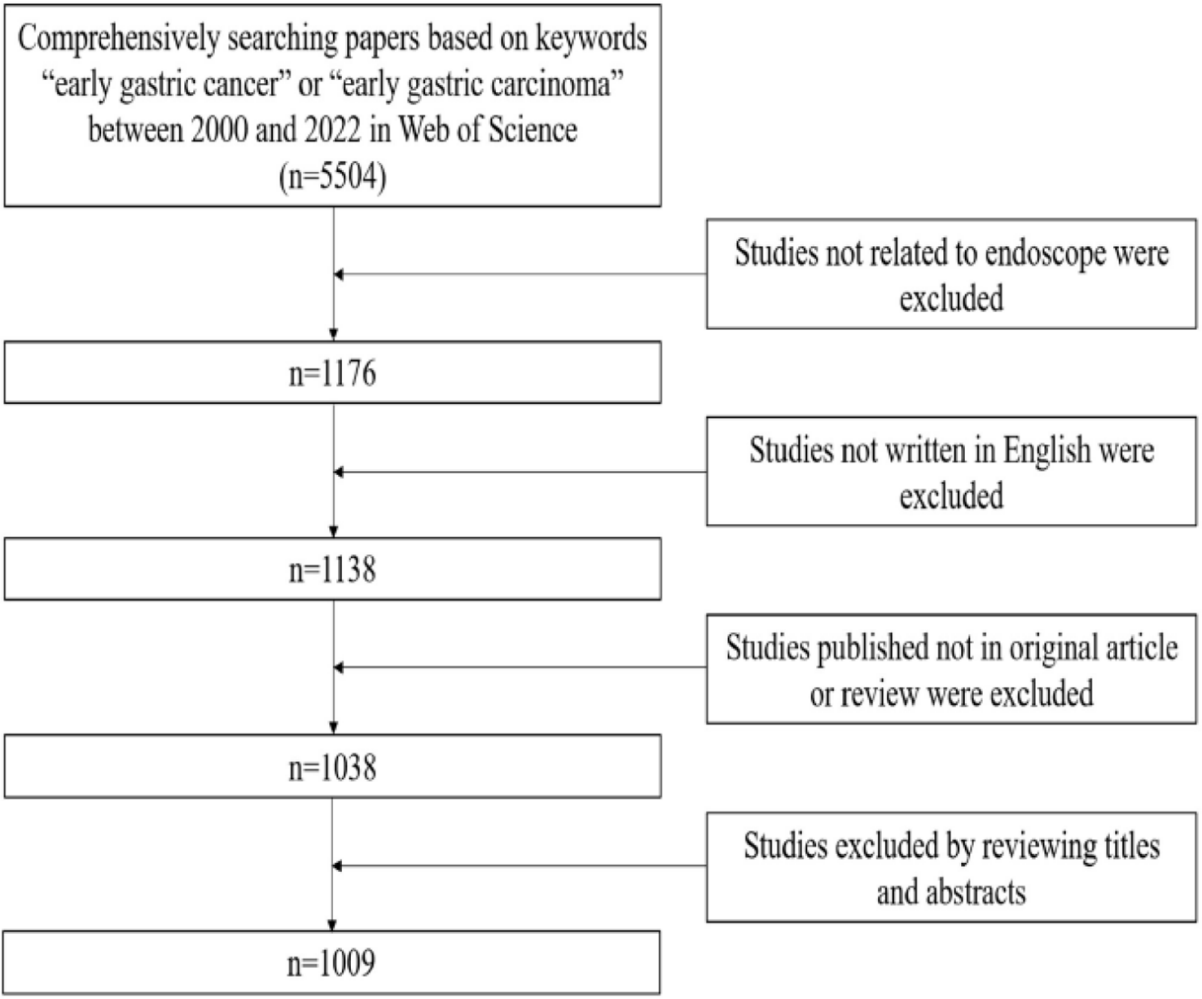
Process of paper selection in the endoscope on the EGC.

We comprehensively searched the WoSCC database to identify relevant papers. Ultimately, a total of 1,009 papers met the eligibility criteria and were included, as shown in **Figure 1**. These papers were written in various countries, institutions, and authors. **Table 1** lists the 10 papers with the highest number of citations (15–24). These 10 papers were cited a total of 4,890 times, representing 19.6% of the total 24,980 citations. The paper titled “Endoscopic mucosal resection for treatment of early gastric cancer,” written by Ono from Japan in 2001 was the most cited paper (1,207 citations) (15).

**Table 1:** Top 10 cited papers in endoscope on EGC.

Rank	Author	Year of publication	Country	Title	Total citations
1	Ono et al. (15)	2001	Japan	Endoscopic mucosal resection for treatment of early gastric cancer	1,207
2	Chey et al. (16)	2007	USA	American college of gastroenterology guideline on the management of Helicobacter pylori infection	846
3	Pimentel-Nunes et al. (17)	2015	Portugal	Endoscopic submucosal dissection: European Society of Gastrointestinal Endoscopy (ESGE) Guideline	690
4	Gotoda et al. (18)	2006	Japan	Endoscopic submucosal dissection of early gastric cancer	508
5	Soetikno et al. (19)	2005	USA	Endoscopic mucosal resection for early cancers of the upper gastrointestinal tract	418
6	Ono et al. (20)	2016	Japan	Guidelines for endoscopic submucosal dissection and endoscopic mucosal resection for early gastric cancer	328
7	Choi et al. (21)	2018	South Korea	Helicobacter pylori Therapy for the Prevention of Metachronous Gastric Cancer	286
8	Sergey et al. (22)	2008	USA	Endoscopic mucosal resection and endoscopic submucosal dissection	229
9	Rosch et al. (23)	2004	Germany	Attempted endoscopic en bloc resection of mucosal and submucosal tumors using insulated-tip knives: A pilot series	206
10	Paspatis et al. (24)	2014	Greece	Diagnosis and management of iatrogenic endoscopic perforations: European Society of Gastrointestinal Endoscopy (ESGE) Position Statement	172

**Figure 2:**
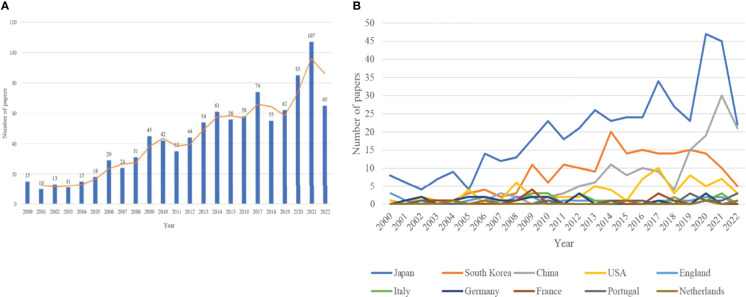
**(A)** The annual number of published papers in endoscope on EGC over the past 20 years. **(B)** The annual trend in publications in endoscope on EGC by country over the past 20 years.

Since the beginning of the 21st century, many papers on the use of endoscopy for EGC have been published. **Figure 2A** shows the increasing trend in the number of publications over the past 20 years. There were three stages in the research trends. The first stage was from 2000 to 2011. At the beginning, between 10 and 20 papers were published each year, but this number continuously increased and reached a maximum of 45 annual publications. However, in 2011, the publication volume dropped to 35. The second stage was from 2012 to 2018. The number of publications exploded and reached 74 in 2017 but decreased to 55 in 2018. The third stage was from 2019 to 2022. At this stage, the increase in publication numbers was apparent, peaking at 107 in 2021. Due to the timing of the current analysis, the 65 publications recorded for 2022 do not represent the whole number for this year.

### Distributions of countries and institutions

These 1,009 papers were published in 48 countries and in 201 institutions. **Table 2** shows the top 10 countries, including six European countries, three Asian countries, and one North American country. These countries published 956 papers in total, representing 94.7% of the total number of publications. As shown in **Figure 2B**, three Asian countries (Japan, South Korea, and China) were highly productive, and they each published more than 100 papers separately over the past 20 years; the total number of publications from these three countries was 785, representing 77.8% of the total number of publications. Japan continued to be the leader in publications (452, 44.8%), followed by South Korea (183, 18.1%) and China (150, 14.9%). Japan also had the highest centrality among countries and institutions. The number of publications in China has generally increased in recent years as well.

**Table 2:** Top 10 countries and institutions involved in endoscope on EGC.

Rank	Country	Number (% of 1,009)	Centrality	Institution	Number (% of 1,009)	Centrality
1	Japan	452 (44.8)	0.38	National cancer center Japan	48 (4.8)	0.19
2	South Korea	183 (18.1)	0.00	Yonsei university	40 (4.0)	0.02
3	China	150 (14.9)	0.15	Fukuoka university	39 (3.9)	0.16
4	USA	76 (7.5)	0.20	Yonsei university health system	35 (3.5)	0.02
5	England	22 (2.2)	0.11	Japanese foundation for cancer research	31 (3.1)	0.04
6	Italy	19 (1.9)	0.03	Seoul national university	31 (3.1)	0.00
7	Germany	21 (2.1)	0.03	University of Tokyo	25 (2.5)	0.03
8	France	14 (1.4)	0.05	Kyoto prefectural university of medicine	24 (2.4)	0.01
9	Portugal	14 (1.4)	0.11	University of Ulsan	23 (2.3)	0.02
10	Netherlands	5 (0.5)	0.02	Jikei University	22 (2.2)	0.11

Centrality is used to evaluate the location of a node (a keyword, country, institution, or author) in a network. Higher centrality reflects greater effects (25). Japan has the highest centrality among countries—which means it contributes a large number of influential papers in this field—followed by the USA, China, and England (15, 26, 27). It has the highest centrality in institutions as well. However, South Korea has a centrality of 0.00, but it had the second highest number of publications. Some European countries, such as Italy, Germany, and the Netherlands, also have low centralities. This suggests that the quality of papers from these countries needs to improve, as increasing the number of publications is not sufficient.

In addition to the difference in the number of published articles, there are also some differences in the research emphasis between eastern and western countries. Western countries, such as the USA, England, and Italy, focus on the etiology, diagnosis, detection, and screening of EGC, while eastern countries, such as Japan, China, and South Korea, pay more attention to the treatment of EGC. However, in recent years, both eastern and western countries have paid increasing attention to the use of artificial intelligence in the field of EGC.

**Figure 3:**
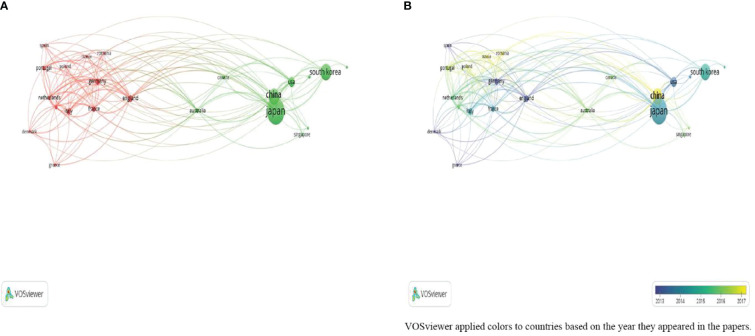
**(A)** Network visualization of countries; **(B)** Overlay visualization of countries.

**Figure 3A** also shows the links between countries. Asian countries (Japan, China, and South Korea) are more closely linked to the USA than European countries (England, Italy, and Germany). Earlier, Germany, England, Spain, and Japan were more concerned with the use of endoscopy for EGC. China, Russia, and Romania were the most recent countries concerned about this topic (**Figure 3B**).

**Figure 4:**
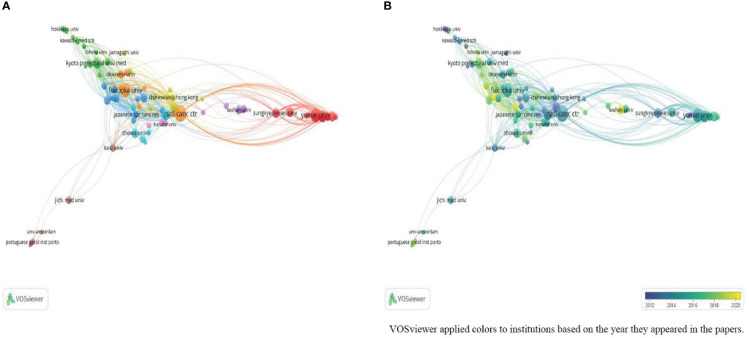
**(A)** Network visualization of institutions; **(B)** Overlay visualization of institutions.

Regarding institutions, the 10 most productive institutions published 318 papers, representing 31.5% of the total number of publications. The National Cancer Center in Japan was the most prolific institution (48, 4.8%), followed by Yonsei University (40, 4.0%), and Fukuoka University (39, 3.9%). The top 10 institutions were all from Asia, including six Japanese and four South Korean institutions. Japanese institutions were tightly connected. The number of Chinese institutions has gradually increased in recent years as well (**Figure 4**).

### Distributions of journals

In total, 224 journals published papers about the use of endoscopy for EGC over the past 20 years. **Table 3** shows the top 10 journals, which accounted for 448 publications, representing 44.4% of the total number of publications. Surgical Endoscopy and Other Interventional Techniques was the most productive journal (77, 7.6%), followed by the World Journal of Gastroenterology (64, 6.3%), and Digestive Endoscopy (61, 6.0%). Each of the top 10 journals published at least 20 papers on the use of endoscopy for EGC. Seventy percent of the 10 journals belong to Q1 or Q2. Among them, Gastrointestinal Endoscopy had the highest impact factor (10.396 in 2021). The Journal Citation Indicator (JCI) is the average Category Normalized Citation Impact (CNCI) of citable items (articles and reviews) published by a journal over a recent three-year period. Among these 10 journals, Endoscopy has the highest JCI (2.47).

**Table 3:** Top 10 journals involved in endoscope on EGC.

Rank	Journal	Impact factor 2021	Number (% of 1,009)	JCI	JCR	Total citations	Average citation
1	Surgical endoscopy and other interventional techniques	3.453	77 (7.6)	1.43	Q2	1,649	21.4
2	World journal of gastroenterology	5.374	64 (6.3)	0.84	Q2	1,306	20.4
3	Digestive endoscopy	6.337	61 (6.0)	1.53	Q2	1,635	26.8
4	Endoscopy	9.776	54 (5.4)	2.47	Q1	4,294	79.5
5	Gastrointestinal endoscopy	10.396	51 (5.1)	1.85	Q1	3,049	59.8
6	Gastric cancer	7.708	42 (4.2)	1.55	Q1	1,261	30.0
7	Journal of gastroenterology and hepatology	4.369	33 (3.3)	0.77	Q2	536	16.2
8	Digestion	3.672	23 (2.3)	0.72	Q3	272	11.8
9	Digestive diseases and sciences	3.487	22 (2.2)	0.65	Q3	284	12.9
10	Hepato gastroenterology	0.792	21 (2.1)	NA	Q4	288	13.7

NA, Not applicable.

### Distributions of authors

In total, approximately 200 authors have published papers on the use of endoscopy for EGC over the past 20 years. **Table 4** lists the top 10 authors (nine Japanese authors and one South Korean author), who published a total of 209 papers, representing 20.7% of the total number of publications. Each of the top 10 authors published more than 15 papers. Oda Ichiro from Japan published the largest number of papers (31, 3.1%) and had the highest H-index (58).

**Table 4:** Top 10 authors involved in endoscope on EGC.

Rank	Author	Country	Number (% of 1,009)	H-index	Centrality
1	Oda, Ichiro	Japan	31 (3.1)	58	0.09
2	Yao, Kenshi	Japan	29 (2.9)	27	0.07
3	Uedo, Noriya	Japan	24 (2.4)	50	0.09
4	Gotoda, Takuji	Japan	21 (2.1)	56	0.06
5	Fujishiro, Mitsuhiro	Japan	20 (2.0)	57	0.10
6	Fujisaki, Junko	Japan	19 (1.9)	28	0.00
7	Hirasawa, Toshiaki	Japan	17 (1.7)	26	0.00
8	Lee, Sangkil	South Korea	16 (1.6)	36	0.00
9	Iwashita, Akinori	Japan	16 (1.6)	34	0.10
10	Nagahama, Takashi	Japan	16 (1.6)	19	0.01

**Figure 5:**
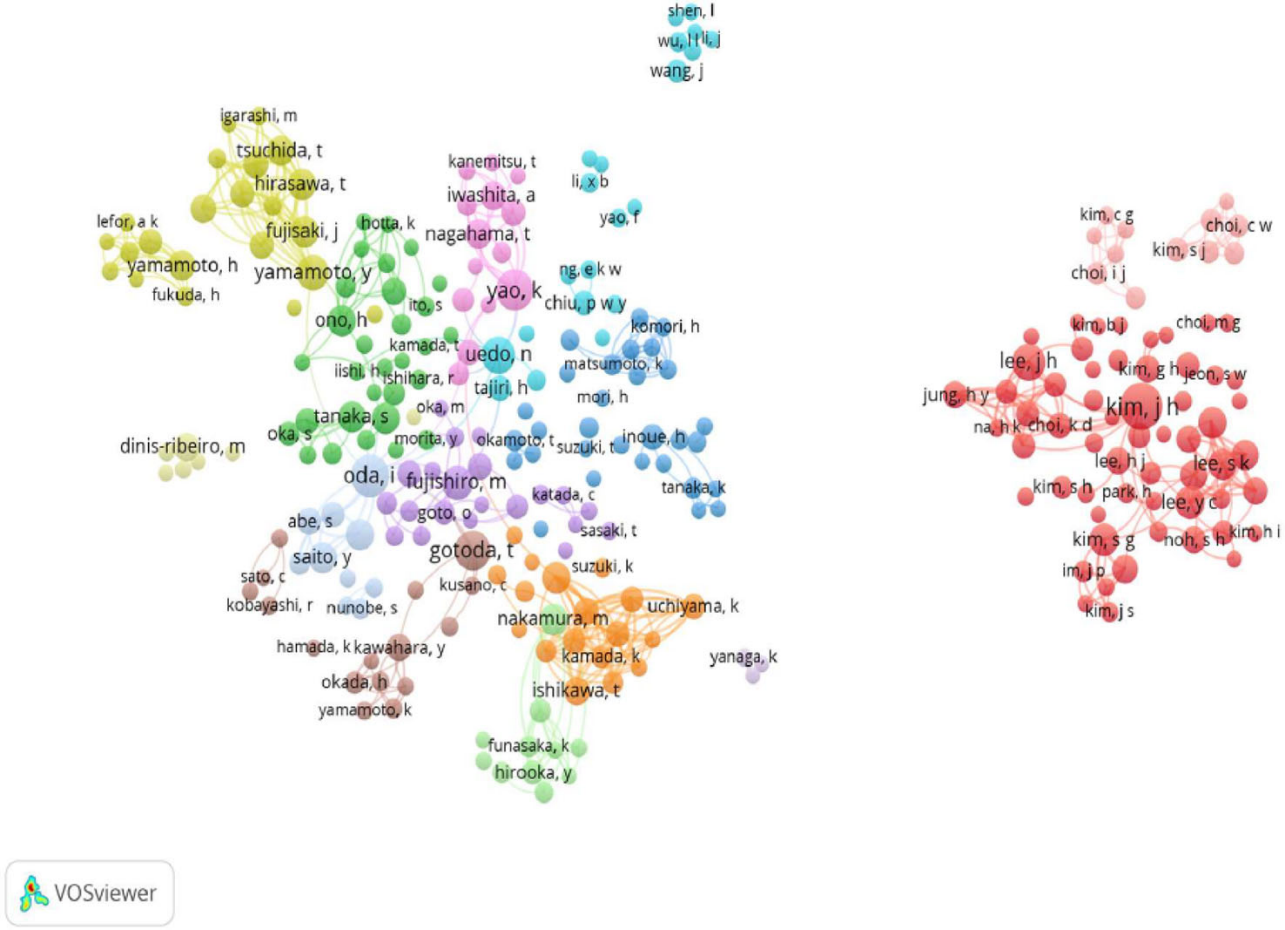
Network map of authors in endoscope on EGC researchers.

In addition, a network map among authors was constructed using VOSviewer software (**Figure 5**). This network map shows the cooperation among authors. Each circle represents an author, and the lines between the circles represent the relationship between authors. The connection network shown in various colors indicates clusters of cooperation among different authors (28, 29). The shorter the link, the stronger the connection between authors is. As shown in **Figure 5**, authors were divided into various colors and connected by links. Japanese authors are closely connected on a small scale, but there is also considerable overall; furthermore, these networks have the largest number of authors. Oda, Ichiro, Yao, Kenshi, Uedo, and Noriya are Japanese authors who were included in the top 10 authors; they are included in this map and have large circles. South Korean authors were also closely connected and had the second-highest number of authors. Chinese authors had the third highest number of authors, but they were not as closely connected as authors in Japan and South Korea. Authors from Japan, South Korea, and China are not very connected.

### Distributions of hotspots and frontiers

Keywords represent the key points or the cores of a paper. Analyzing keywords and their connections helps researchers track knowledge development and find direct and popular topics (25). Recently, co-occurrence analysis has been commonly used to find hotspots and frontiers in specific fields, and it has proven to be significant in academic research. In the co-occurrence map, each circle represents a keyword, and the links between the circles represent connections. A larger circle and a thicker link represent a higher frequency of occurrence and a stronger connection. Different colors on the map represent different clusters. Items of the same color are in the same cluster.

**Figure 6:**
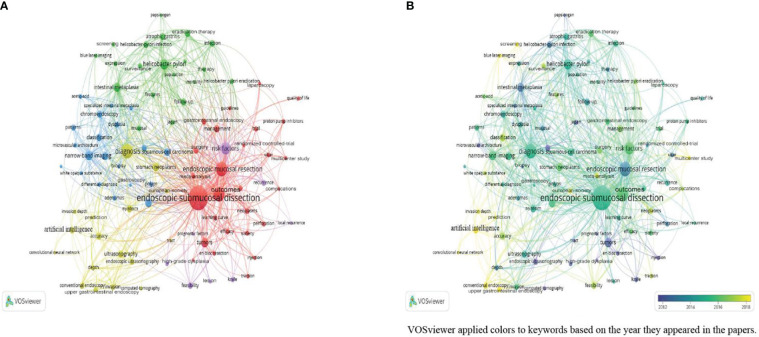
**(A)** Network visualization of keywords; **(B)** Overlay visualization of keywords.

Of the included 1,009 papers, 2,082 keywords exist. Among them, 92 items were used more than 15 times in papers. The most used items were “endoscopic submucosal dissection” (448 times), “diagnosis” (168 times), “endoscopic mucosal resection” (166 times), “outcomes” (164 times), and “risk factors” (122 times). **Figure 6** shows the network and overlay visualizations of keywords. They were mainly divided into four clusters: the green cluster represents “etiology,” the yellow cluster represents “endoscopic diagnosis,” the red cluster represents “endoscopic therapy,” and the blue cluster represents “others (reviews or case reports)” (**Figure 6A**).

As shown in **Figure 6B**, researchers mainly focused on etiologies such as “*H. pylori*,” “atrophic gastritis,” and “intestinal metaplasia” in the early years. Over time, the focus shifted to endoscopic therapy and novel endoscopic diagnosis methods such as “artificial intelligence” (AI) and “convolutional neural networks” (CNNs).

**Figure 7:**
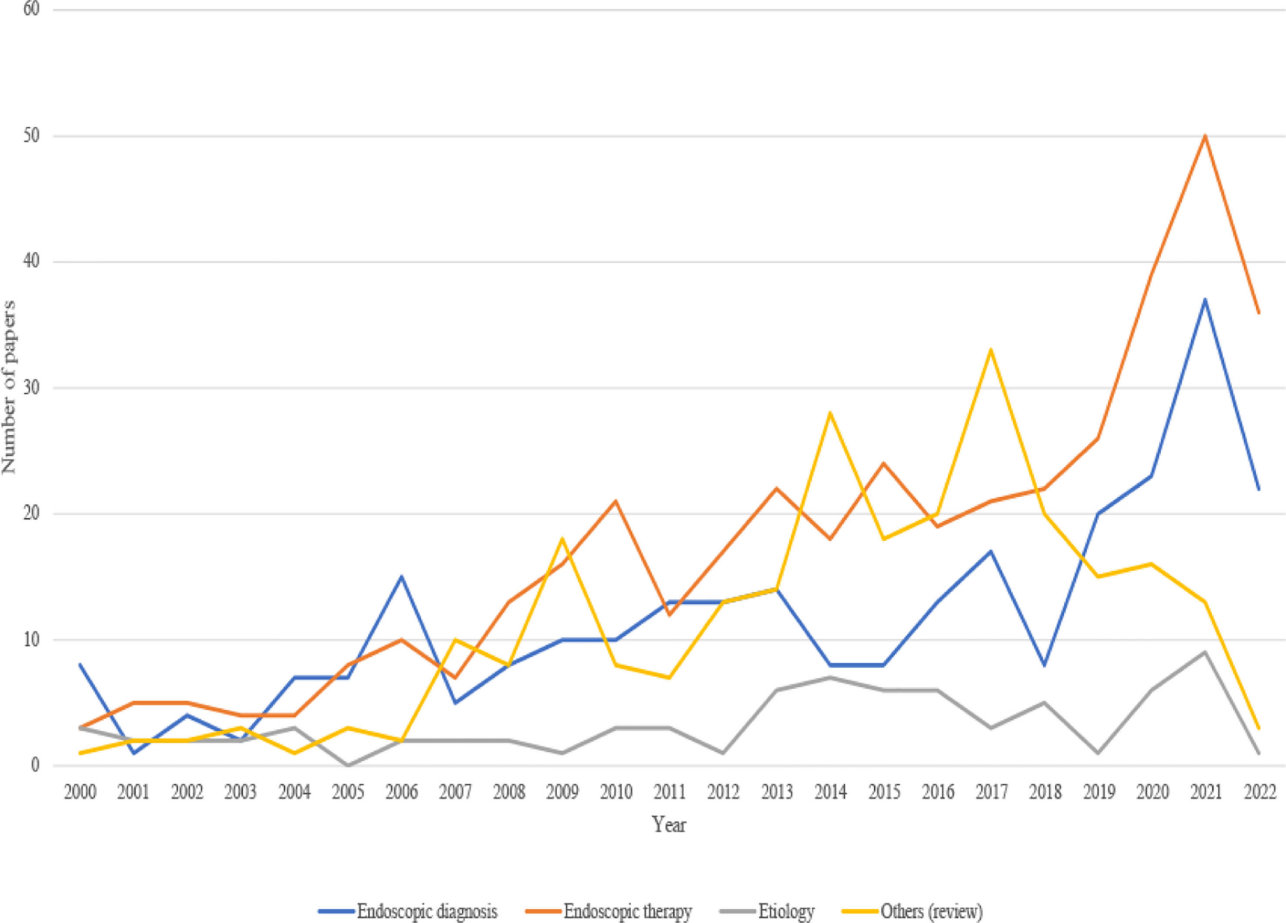
Annual research trend of publications of endoscopes on EGC over the past 20 years.

We divided papers into the above four topics according to the main research contents, themes, and types of manuscripts as well: “etiology” topic (76), “endoscopic diagnosis” topic (271), “endoscopic therapy” topic (402) and “others (reviews or case reports)” topic (260). **Figure 7** shows research trends for each topic over the past 20 years. Papers related to “endoscopic diagnosis,” “endoscopic therapy,” and “others (reviews or case reports)” continuously increased. After 2017, papers on the “others (reviews or case reports)” topic decreased. In contrast, the number of papers on the “etiology” topic remained unchanged.

**Figure 8:**
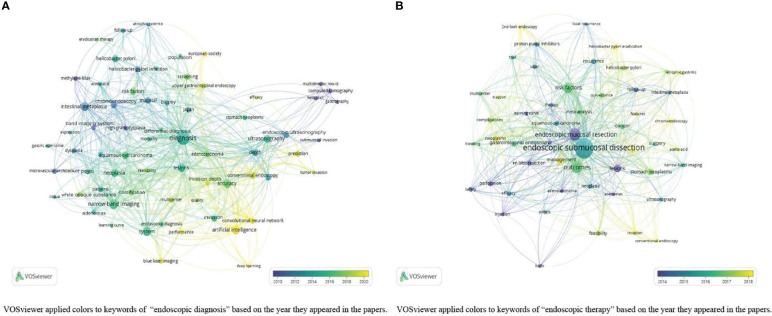
**(A)** Overlay visualization of keywords in topic 1 “endoscopic diagnosis”; **(B)** Overlay visualization of keywords in topic 2 “endoscopic therapy”.

We also performed additional keyword analysis on topics 1 (“endoscopic diagnosis”) and 2 (“endoscopic therapy”) for further analysis using VOSviewer. For papers classified into topic 1 (“endoscopic diagnosis”), there were 808 total keywords, and 67 items were used more than five times. To identify the research, we excluded the research keywords “early gastric cancer” and “endoscopy.” **Figure 8A** shows the overlay visualization of topic 1 (“endoscopic diagnosis”). In the early stages, the items “intestinal metaplasia” (which appeared 25 times), “*Helicobacter pylori* infection” (which appeared 13 times), and “high-grade dysplasia” (which appeared nine times) appeared. Then, the items “narrow-band imaging” (30 times), “classification” (used 13 times), and “endoscopic diagnosis” (eight times) appeared. The latest trend showed that the keywords “AI” (used 23 times), “CNN” (13 times), and “deep learning” (used five times) would be the hotspots and frontiers in the coming years.

In the analysis of topic 2 (“endoscopic therapy”), 52 keywords were used more than 10 times among all 1,318 keywords (**Figure 8B**). Items “endoscopic mucosal resection” (90 times), “perforation” (18 times), and “en bloc resection” (16 times) were in the early stage, followed by the items “endoscopic submucosal dissection” (240 times), “outcomes” (88 times), and “surveillance” (16 times) over time. The keywords “*Helicobacter pylori* eradication” (used 11 times) and “2nd-look endoscopy” (used eight times) will be investigated the most in the future.

## Discussion

In this study, bibliometric and visual analyses were performed to investigate the global research trends in endoscopy on EGC over the past 20 years. There was an increase in cumulative publications, especially in Asian countries. AI and deep learning, second-look endoscopy, and management are hotspots in endoscopic diagnosis and endoscopic therapy in current research.

EGC is an invasive gastric cancer that occurs in the gastric mucosa and submucosa layer, with or without lymph node metastasis, and it affects patient prognosis (30). In the early stages, due to the limited development of endoscopy, gastric cancer is often detected in the advanced stage, so surgical treatments are the only options. However, complications of surgery include perforation, bleeding, and infection. Clinicians have gradually noticed the applications of the use of endoscopy for EGC with its development. Since entering the 21st century, researchers have focused on the use of endoscopy for EGC, such as endoscopic diagnosis and therapy, and applied the research results to the clinic. This helps patients with early detection and diagnosis of gastric cancer, prolong life, and improve quality of life (31, 32).

Bibliometric analysis was used to explore the current situation and characteristics of specific fields and confirm the hotspots by visualizing the bibliometric data (33, 34). WoSCC includes more scientific publications and provides an overall data source for bibliometric software. Hence, WoSCC is the most used database for bibliometric analysis. In this study, we analyzed the bibliometric data on endoscopy and EGC over the past 20 years. The number of publications increased and reached a maximum of 107 in 2021. In addition, 70% of the top 10 papers were published in journals in Q1 or Q2. These findings reflect the fact that an increasing number of scientists have noticed the importance and necessity of the use of endoscopy for EGC (30, 35, 36). The top three countries in terms of the number of publications were Japan, South Korea, and China, which are all Asian countries. The incidence and prevalence of gastric cancer are characterized by complex geographical changes and are highest in Central and Eastern Asia (1, 37). The Correa hypothesis suggests that people exert considerable effort in high-risk countries to delay gastric carcinogenesis (25). Asian countries attach greater importance to the use of endoscopy for EGC than European countries. This is consistent with the epidemiological status and hypothesis. According to the number of publications, China has made considerable progress in recent years in this field, but there is still a gap between China, Japan, and the USA in terms of highly cited papers, thus requiring further development (15, 16).

Network and overlay visualizations reveal the frequency and connections among keywords, countries, institutions, and authors, which visualize data (38). Asian countries were more connected than European countries. This is possibly due to the differences in the prevalence of EGC (39). Japanese authors and institutions are connected more closely with each other than in other countries. Japan also has authors who have published the largest number of papers and had the highest centrality. This suggests that tight connections among authors, institutions, countries, and regions are essential for the development of academic research. Close connections contribute to promoting scientific progress.

Keywords are the core and central of papers. They summarize and represent the main content, academic ideas, and final conclusions. By analyzing the keywords of a large number of papers, researchers can generalize the current status and situation (29). Of the included papers, 2,082 keywords exist, and 92 items were used more than 15 times in the papers. From the point of view of keywords and time evolution, endoscopic diagnosis and therapy are hotspots on EGC. The number of publications on different topics also shows that. Recently, an increasing number of researchers have focused on novel technical methods such as AI and CNN with the development of endoscopic techniques (6, 40–42). The excellent veracity and sensitivity of AI were proven. The results indicate that we should focus on novel endoscopic techniques in the future.

Regarding endoscopic diagnosis, AI, CNN, and deep learning were hotspots. In recent years, AI in the field of endoscopy has been a new area of interest. Hirasawa et al. reported that the sensitivities of using AI for white light endoscopy and magnifying narrow band imaging were 92% and 97%, respectively (43). A meta-analysis written by Jiang stated that the sensitivity and specificity of AI in detecting EGC were not low, at 86% and 93%, respectively (44). Additionally, some researchers have also suggested that, compared to experienced endoscopists, AI is neither inferior nor superior (45). Wu et al. invented a novel system to diagnose EGC called ENDOANGEL, which was promising in detecting EGC (46). In conclusion, although the results of our study show that recent studies have focused on AI, CNN, and some other novel endoscopic techniques in diagnosing EGC, it is important to correctly define the roles of AI and the balance of AI between clinical endoscopists.

ESD and EMR are commonly used for EGC treatment in the clinic, while EGC patients have a high risk of complications, including developing synchronous and metachronous gastric lesions (MGLs) after curative endoscopic therapy (47). The bibliometric analysis performed herein using VOSviewer software showed that researchers were mainly concerned with 2nd-look endoscopy and management after endoscopic therapy. Ortigao et al. (48) discussed how to manage EGC after endoscopic therapy to improve quality of life. Sekiguchi (49) also evaluated better methods of management for elderly EGC patients in Japan. These findings imply that reexamination and management after endoscopic therapy are as beneficial as the endoscopic process itself.

In addition to AI, deep learning, and other hotspots, we found that *H. pylori* eradication, 2nd-look endoscopy and management, gastric cancer screening, and the cost-effectiveness of endoscopy have gradually become hot research topics in recent years through bibliometric analysis. Wang et al. (50) found that eradication of *H. pylori* could prevent postoperative recurrence of EGC and prolong the overall survival of EGC patients. Shichijo et al. (51) showed that timely eradication of *H. pylori* also increased the risk of developing gastric cancer. Therefore, timely and effective detection and management of high-risk groups, as well as 2nd-look endoscopy, are necessary. In addition, the cost-effectiveness of endoscopy should be considered. The use of appropriate risk screening and prediction systems, such as the new gastric cancer screening scoring system (NGCS), can provide an important basis for decision-makers to formulate and optimize EGC prevention and control policies and save on health care costs (52).

There are some limitations to this study and various factors influencing the research results as well. First, we only retrieved papers from WoSCC, which may lead to incomplete searches. Second, we only included papers published in English, and thus, some papers published in other languages may be missed. Third, selection bias cannot be ignored, although two persons reviewed and screened the initial papers. A search based on keywords and abstracts means that a small number of manuscripts dealing with EGC may not be identified. Finally, since many authors have the same initials and some keywords are expressed differently, even though we have standardized them, there may still be bias. Therefore, the results and conclusions should be considered considering all the limitations and influencing factors mentioned above. Even so, this study still discussed the research trends in endoscopy on EGC over the past 20 years through bibliometric analysis in a comprehensive way to some extent, which is helpful to understand the development of endoscopy on EGC and the possible hotspots in the future.

The current study analyzed global research trends in the use of endoscopy for EGC. The results show that EGC research is gaining traction, with an increasing trend in the average number of papers published each year. Our study shows that research on EGC focuses on AI, CNN, and deep learning for the early screening and diagnosis of the disease. In addition, it is of great significance to apply the research results in the clinic in the future. The results of this study will help readers understand the research status of EGC, provide research direction and ideas for clinic researchers, and provide support for future clinical trials to help improve the quality of life for patients with EGC.

## Conclusions

In conclusion, this study analyzed global research trends in the use of endoscopy for EGC over the past 20 years. The results revealed an increase in cumulative publications, especially in Asian countries. The use of endoscopy for diagnosis and therapy was the focus of publications. AI, CNN, and deep learning were hotspots in endoscopic diagnosis. Reexamination and management after endoscopic therapy were necessary and essential as well.

## Data availability statement

Publicly available datasets were analyzed in this study. This data can be found here: The datasets generated for this study can be found in DOI: https://data.mendeley.com/datasets/h4v878vgks/1.

## Author contributions

Conceptualization, SL and NZ. Methodology, SL. Software, SL and YH. Validation, SL and YH. Formal Analysis, SL. Investigation, SL and NZ. Resources, NZ. Data Curation, SL. Writing—Original Draft Preparation, SL. Writing—Review and Editing, SL and NZ. Visualization, SL. Supervision, PL. Project Administration, NZ and PL. Funding Acquisition, PL. All authors contributed to the article and approved the submitted version.
